# A Case of Tumor Embolism to the Lower Extremity Artery Caused by Pulmonary Sarcoma With Left Atrial Invasion

**DOI:** 10.1002/ccr3.73184

**Published:** 2026-07-20

**Authors:** Tatsuhiko Uno, Kyoichi Kaira, Ou Yamaguchi, Kosuke Hashimoto, Atsuto Mouri, Hisao Imai, Hiroshi Kagamu

**Affiliations:** ^1^ Department of Respiratory Medicine, Comprehensive Cancer Center, International Medical Center Saitama Medical University Saitama Japan

**Keywords:** atrial invasion, Fogarty catheter, lower extremity artery, pulmonary sarcoma, tumor embolism

## Abstract

Arterial tumor embolism is a fatal complication of human neoplasia. Here, we report a case of an arterial tumor embolism in the lower limb that occurred after chemoradiotherapy for primary pulmonary sarcoma. Initially, the primary tumor had invaded the left atrium, originating from the right pulmonary vein.

## Key Clinical Message

Arterial tumor embolism is a fatal complication of human neoplasms. Pulmonary artery sarcoma is one of the neoplasms causing arterial tumor embolism. Acute lower limb ischemia resulting from arterial tumor embolism due to pulmonary sarcoma rarely occurs; however, clinicians should be alert to this severe phenomenon.

## Graphical abstract

Arterial tumor embolism is a fatal complication of human cancers. A 64‐year‐old male experienced arterial tumor embolism in the lower limb that developed after chemoradiotherapy for primary pulmonary sarcoma.

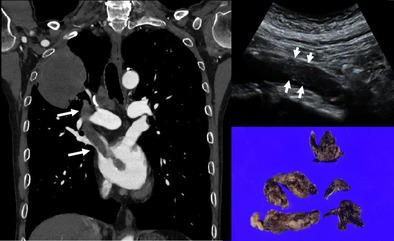

## 1 Clinical Presentation

**FIGURE 1:**
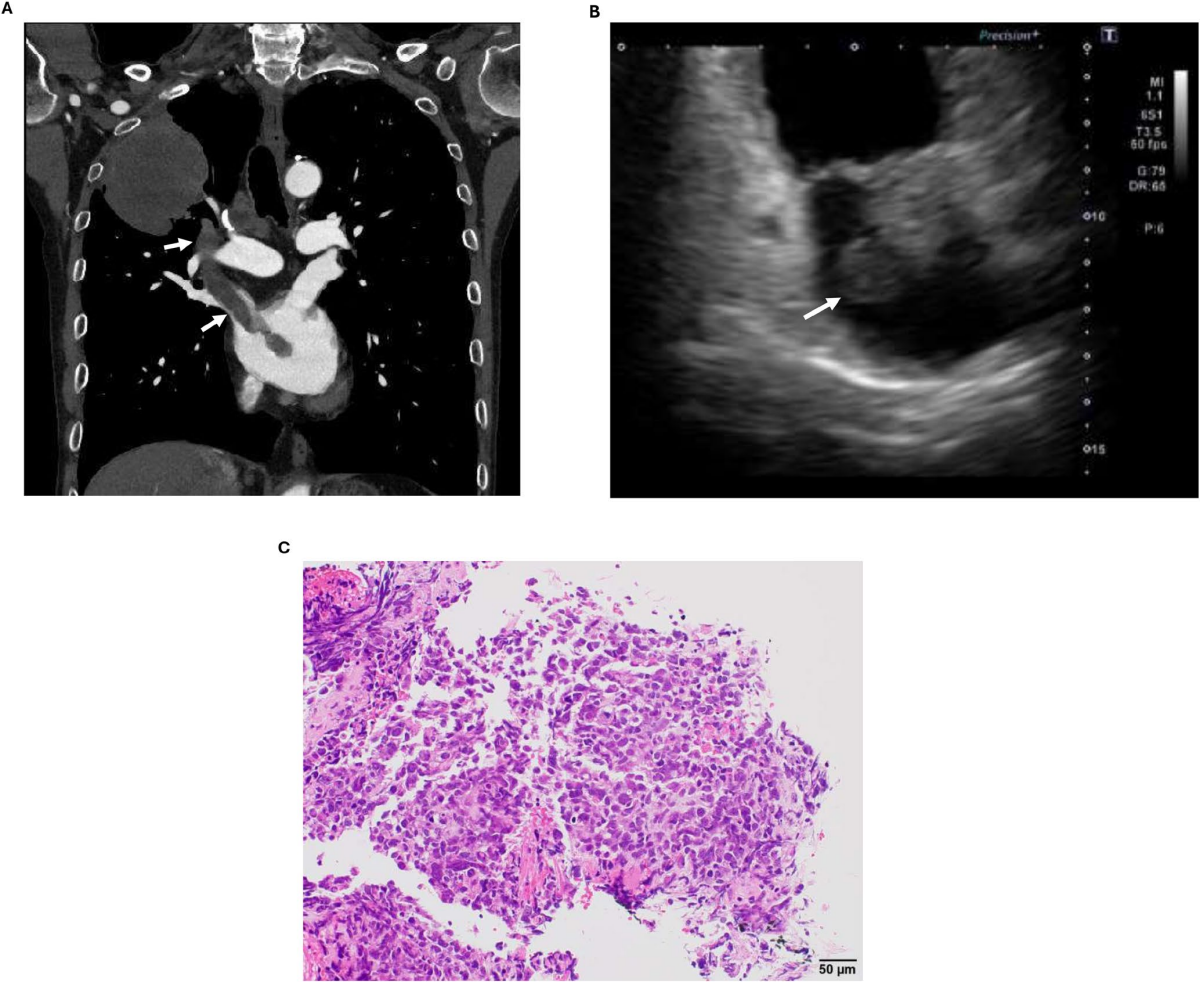
Coronal chest computed tomography shows massive tumor accompanied by a contrast‐filling defect in the left atrium originating from the right pulmonary vein (white arrows) (A). Cardiac echocardiography shows a mobile mass shadow in the left atrium (B). Histological examination reveals pulmonary sarcoma (C).

**FIGURE 2:**
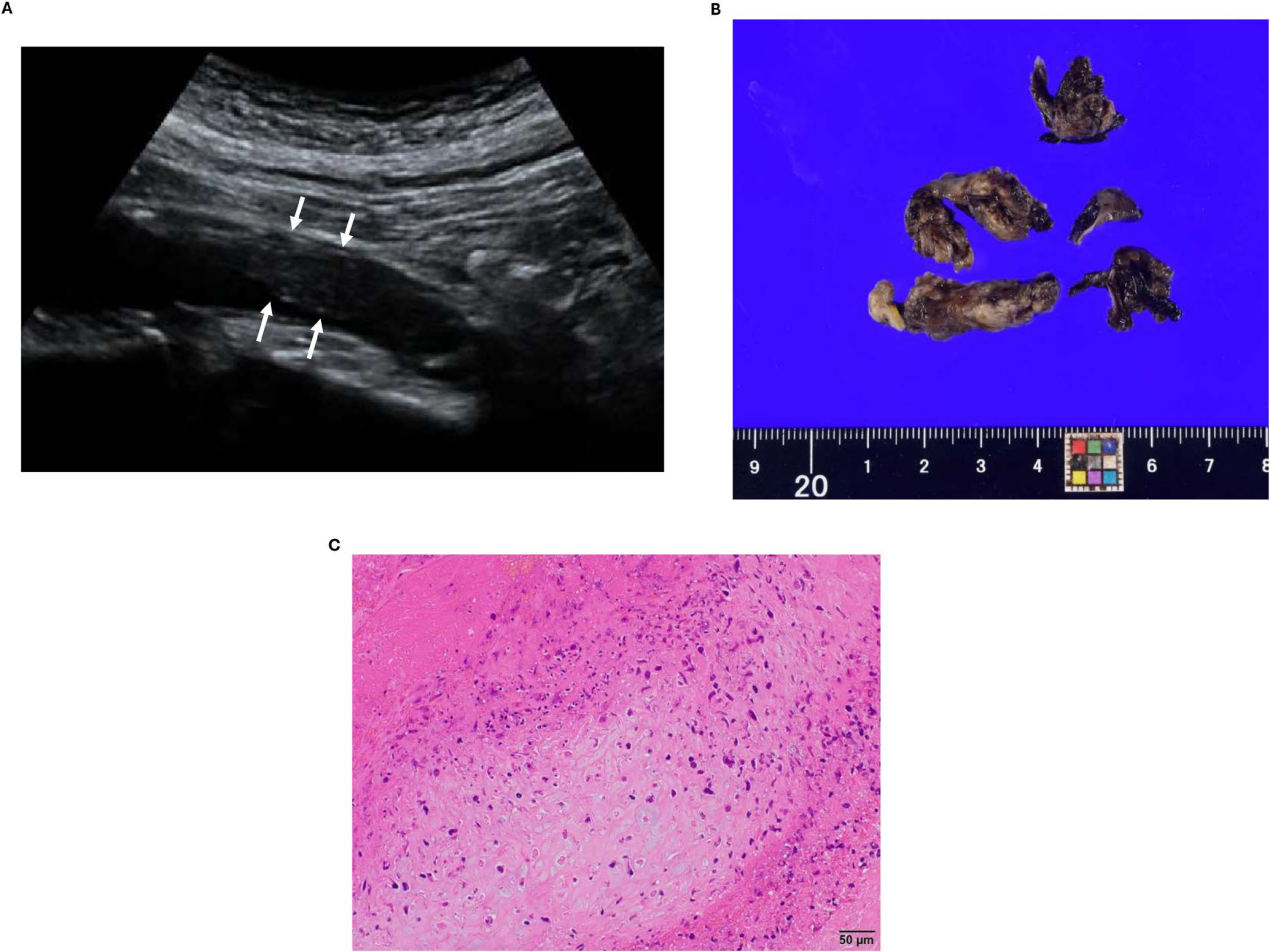
Lower extremity arterial ultrasound shows tumor embolism to the lower extremity artery (white arrows) (A), Embolectomy using a Fogarty catheter indicates tumor embolism (B). Histological examination shows pulmonary sarcoma metastasis (C).

A 64‐year‐old man with a history of smoking presented with vertigo. Chest computed tomography (CT) revealed a massive tumor with a contrast‐filling defect in the left atrium originating from the right pulmonary vein (Figure 1A). Cardiac echocardiography revealed a mobile mass shadow in the left atrium (Figure 1B). Diagnostic transbronchial biopsy revealed stage IIIB pulmonary sarcoma (Figure 1C). After initiating heparinization, chemoradiotherapy with carboplatin and paclitaxel was administered. After 41 days, the patient experienced pain in the right lower limb. Lower extremity arterial ultrasonography revealed a tumor embolism in the lower extremity artery (Figure 2A). Therefore, embolectomy using a Fogarty catheter was performed, and histological examination revealed metastasis to the pulmonary sarcoma (Figure 2B,C). Acute lower limb ischemia resulting from an arterial tumor embolism due to pulmonary sarcoma was diagnosed. Arterial tumor embolism was identified in 0.3% of 887 cases of arterial emboli [1]. Most patients with arterial tumor embolisms are known to have primary lung cancer [2]. Embolectomies using Fogarty catheters are essential for the resection of different diagnoses and treatments of arterial tumor embolisms in addition to anticoagulation. Although the common sites of embolism are lower extremity veins, our case was considered as a tumor thrombus from a synchronous pulmonary artery sarcoma, not local invasion. In our case, however, the therapeutic shrinkage by chemoradiotherapy may cause cardiovascular complications such as tumor embolisms. The relationship between chemoradiotherapy and tumor embolism would support the understanding of treatment‐related abnormality.

## Author Contributions

**Ou Yamaguchi:** data curation. **Kosuke Hashimoto:** data curation. **Hisao Imai:** data curation, supervision. **Hiroshi Kagamu:** supervision, resources, writing – review and editing. **Tatsuhiko Uno:** conceptualization, methodology, writing – review and editing. **Kyoichi Kaira:** conceptualization, methodology, writing – review and editing. **Atsuto Mouri:** data curation.

## Funding

The authors have nothing to report.

## Ethics Statement

All procedures involving human participants were performed in accordance with the ethical standards of the institutional and/or national research committees, and the 1964 Declaration of Helsinki and its later amendments or comparable ethical standards.

## Consent

The authors declare that written informed consent was obtained for the publication of this manuscript and the accompanying images using the consent form provided by the journal.

## Conflicts of Interest

The authors declare no conflicts of interest.

## Data Availability

All data generated or analyzed during this study are included in this article. Further inquiries can be directed to the corresponding author.
